# Propagule size and structure, life history, and environmental conditions affect establishment success of an invasive species

**DOI:** 10.1038/s41598-018-28654-w

**Published:** 2018-07-09

**Authors:** Michael A. Tabak, Colleen T. Webb, Ryan S. Miller

**Affiliations:** 1 Center for Epidemiology and Animal Health, United States Department of Agriculture – Animal & Plant Health Inspection Service, 2150 Centre Ave, Bldg B, Fort Collins, CO 80526 USA; 2 Department of Biology, Colorado State University, Fort Collins, CO 80523 USA

## Abstract

Population dynamics of species that are recently introduced into a new area, e.g., invasive species and species of conservation concern that are translocated to support global populations, are likely to be dominated by short-term, transient effects. Wild pigs (*Sus scrofa*, or wild boar) are pulsed-resource consumers of mast nuts that are commonly introduced into new areas. We used vital rate data (i.e., survival and fecundity) for wild pigs in Germany under varying forage conditions to simulate transient population dynamics in the 10-years following introduction into a new environment. In a low forage environment (i.e., conditions similar to their native range), simulated wild pig populations maintained a stable population size with low probability of establishment, while in environments with better quality forage (i.e., conditions similar to parts of their invasive range), high juvenile fecundity and survival facilitated rapid population growth and establishment probability was high. We identified a strategy for simulating population dynamics of species whose reproduction and survival depend on environmental conditions that fluctuate and for predicting establishment success of species introduced into a new environment. Our approach can also be useful in projecting near-term transient population dynamics for many conservation and management applications.

## Introduction

The number of individuals and frequency with which they are released into a new area, termed “propagule pressure,” has long been considered an integral component of the establishment success of newly arrived species^1–3^. There are two main reasons why propagules are intentionally released into new areas. Threatened and endangered species, especially island endemics, are translocated to increase their global population size and to place individuals in locations where they will have a high probability of success^4,5^. Additionally, some invasive species which are valuable for recreation purposes (i.e., hunting and fishing) are moved into new locations to augment or create new populations for recreation. For example, rainbow trout are often moved into new watersheds to support the fishing industry^6^, and ungulates are moved across county, state, and national borders for hunting^7,8^. Sometimes these introductions are not conducted by management agencies, but by individuals acting illegally^9^. In both of these types of situations, managers must be able to predict the probability of population establishment. Once propagules have been introduced into a new location, their success in establishment depends on three main factors: propagule pressure, environmental conditions, and the species’ life history^3^. In order to effectively manage newly introduced species it is important to understand how these factors interact and contribute to the probability of establishment in the first generations following introduction.

There is a well-established gradient between fast- and slow life history strategies. Species with slow life histories (e.g., humans) put more energy into producing few, large offspring with high probability of survival, while species with fast life histories (e.g., rats) reproduce rapidly and produce many offspring^1,10,11^. Debate exists about which end of the slow-fast gradient is most effective in leading to establishment of newly introduced species^12^, though invasive species (e.g., weedy plants) typically take advantage of fast life histories^13,14^. The speed of a species’ life history affects the propagule age structure (i.e., the proportion of individuals in each age class in the initial population) that is most conducive to growth. Typically for slow strategies, adults have higher elasticity, as growth rate varries more with changes in adult survival and fecundity, and increasing adults in the propagule is expected to allow a population to grow in the short term. Younger age classes generally have higher elasticity in species with fast life histories, and increasing the number in the younger age classes is expected to yield faster growth. These theoretical expectations have important potential consequences for invading species. If a species with a fast strategy is introduced into a new, favorable environment, the population is expected to grow faster when more juveniles are in the initial propagule, as younger age classes have higher elasticity in such species^15,16^.

The invasive wild pig (*Sus scrofa*, or wild boar/feral swine) is a successful and extremely destructive ungulate species in its invasive range that includes all continents except Eurasia, where it is native, and Antarctica, where it is absent^17,18^. Wild pigs threaten wildlife, damage agriculture, and spread disease^19,20^. Nevertheless, pigs are released into new locations to support recreational hunting populations^9,21^ and as both intentional release and unintentional escape from domestic pig farms^22,23^. Since these “founding populations” are often selected and moved by humans, they might be large distances from other wild pig populations, and individuals might be proportioned randomly into age classes.

Wild pigs evolved as pulsed resource consumers - species dependent on resources that are occasionally available at high levels^24^ - whose survival and fecundity, or “vital rates,” are much higher in favorable forage conditions^25^. They evolved this type of behavior to maximize population growth in response to the boom and bust cycle of mast trees^26^, but they currently also thrive in environments that lack mast trees, as they substitute other food sources for mast nuts^27–30^. Wild pigs have evolved to be dietary generalists, feeding on a variety of food sources ranging from fungi to carrion^19,31^. Their evolution to dietary generalism has facilitated the expansion of wild pigs into continents (e.g., Australia) and regions (e.g., southwestern USA) where mast trees are absent^32–34^. Nevertheless, their preferred dietary source is mast nuts when available, and they tend to maximize intake during mast^35,36^. The link between fecundity and forage quality is in part due to reproductive maturity being linked more tightly to weight than age, as juveniles that can reach approximately 30-kg faster (i.e., due to abundant forage) will begin reproducing earlier^7,37^. Additionally, females exposed to more nutritious diets experience increased ovulation and implantation rates^38,39^. Similarly, agriculture has been associated with high reproductive success and high density in both their native and invasive ranges^18,27,40^, as wild pigs consume nutrient-rich crops, a food source that can replace mast in their diets^41,42^. Therefore, their establishment success following introduction may depend strongly on both the frequency of mast and the presence of agriculture in the environment. Demographics models are valuable tools for evaluating establishment success under varying environmental conditions.

A common and traditional approach to demographic modeling estimates the population size at the next time step as a function of the current population size times the growth rate^43,44^. These methods often assume that populations are at equilibrium with a stable age structure and exhibit asymptotic growth^43–46^; such methods also ignore the potential for irruptive population dynamics following perturbations^47–49^. Furthermore, when individuals of a species are introduced into a new location, the population is unlikely to meet equilibrium assumptions and may exhibit large increases or declines in population size^50^. Since vital rates vary among age classes, when a population’s age structure is different than the stable age structure, it is expected to exhibit transient dynamics; population growth rate will be influenced by both age structure and the vital rates and could be greater or less than asymptotic population growth rate^51–58^. Therefore, these transient effects may be important for understanding population dynamics of newly introduced populations^50,56^. Explicitly modeling transient dynamics can provide insights into the effects of altering age structure and the resulting changes in population size over the short-term^59^. Recent work has found that greater than 50% of population dynamics can be the result of transient effects^60,61^, and transient dynamics are often influenced by environmental conditions^62^. Despite the importance of transient dynamics, the inclusion of these effects in demographic models for animal species is still rare, particularly for invasive mammals^50,63^.

We modeled the transient population dynamics of wild pigs under different introduction scenarios, i.e., different propagule sizes, and under different environmental scenarios, i.e., mast conditions approximating what they might experience in their native and invasive ranges and in areas with agricultural subsidy. We focused on the effect of mast on wild pig population dynamics, as mast trees are abundant in a large portion of their range, especially North America and Europe, and the cyclical nature of masting is likely to lead to transient dynamics in animals that depend on them^64^. Therefore, our models will be most relevant on these continents. Nevertheless, our approach is relevant for any species whose vital rates change based on fluctuating environmental conditions, including wild pigs on other continents^40,65^. We sought support for Bieber & Ruf’s finding that since wild pigs are pulsed resource consumers, they would be able to take advantage of both ends of the fast-slow life history spectrum^25^. Specifically, when resources are scarce they would be able to sustain populations by taking advantage of high adult survival and when resources are plentiful, higher juvenile fecundity would facilitate rapid growth. By evaluating the effect of age structure in the propagule on growth, we were able to evaluate this hypothesis. Simulations lent support to this hypothesis, as we identified a useful approach for predicting short-term dynamics of founding populations. We discuss these findings with respect to the fast/slow life history continuum in invasive species and demonstrate additional applications of our approach.

## Results

### Summary of simulations

We used population projection matrix (PPM) models to simulate populations of wild pigs for ten years following introduction of propagules into simulated environments. Three types of environment-specific time series were simulated based on forage conditions: (1) an environment with one common mast tree species, (2) an environment with a community of mast trees, and (3) an environment with agricultural subsidy. In each simulated environment, different proportions of good, poor, and intermediate mast years were used to create time series.

### Population dynamics and establishment

**Figure 1:**
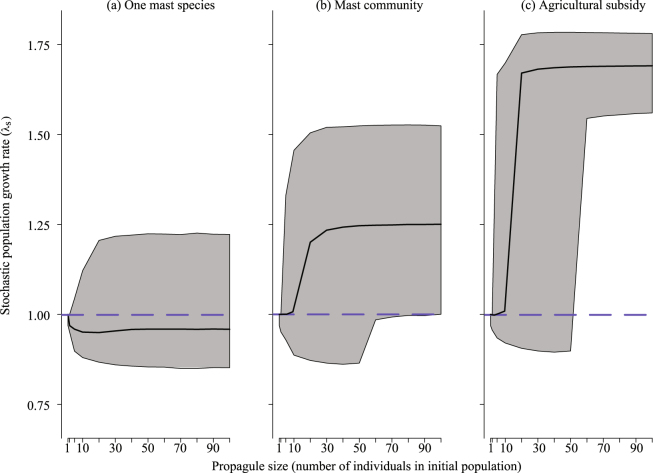
Stochastic population growth rate (*λ*_s_) depended on forage and propagule size. In an environment with one masting tree species (**a**), *λ*_s_ was relatively unaffected by propagule size, while *λ*_s_ increased with propagule size in the environments with a masting community (**b**) and with agricultural subsidy (**c**). In the latter two environments, there was a dramatic increase in *λ*_s_ as propagule size increased above 50 individuals, so that with a propagule size of ≥60, the entire CrI was >1. Solid lines represent medians and shaded areas represent 95% CrIs across 10^6^ realizations. The dashed line represents a static population (neither growing nor decreasing; *λ*_s_ = 1).

Stochastic population growth rate (*λ*_s_) was greater in environments with more mast (Fig. 1). In the environment with one mast species, *λ*_s_ was relatively constant as propagule size increased, with median *λ*_s_ slightly less than one (Fig. 1a). In the environments with a community of mast trees and with agricultural subsidy, median *λ*_s_ increased with propagule size, and eventually stabilized (Fig. 1b,c). In both of these environments, *λ*_s_ increased dramatically beyond a propagule size of 50 individuals; with a propagule size ≥60, the entire credible interval (CrI) for *λ*_s_ was greater than one. Therefore, we set the threshold for population establishment as a growing population (*λ*_s_ > 1) with at least 60 individuals after 10 years.

**Figure 2:**
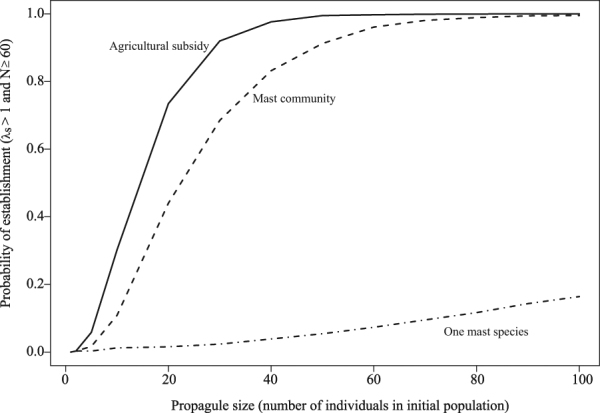
Probability of establishment as a function of propagule size in three different forage environments. Probability of establishment was low for all propagule sizes in the environment with one mast species. In the environments with a mast community and with agricultural subsidy, probability of establishment increased with propagule size and stabilized at >90% probability of establishment.

**Figure 3:**
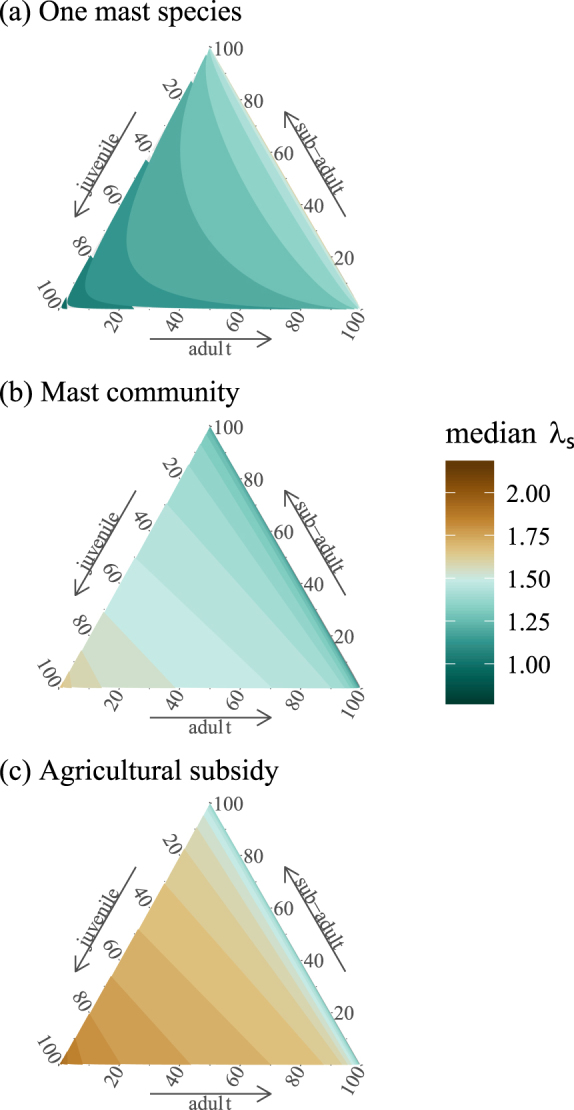
Ternary plots depicting stochastic population growth rate (*λ*_s_) as a function of propagule age distribution. The proportion of individuals in each age class in the propagule increase along the respective axis. In the environment with one mast species (**a**), *λ*_s_ was highest when there were more adults and fewer juveniles in the propagule. Meanwhile, in environments with a mast community (**b**) and with agricultural subsidy (**c**), *λ*_s_ was highest when there were more juveniles in the propagule. This relationship mimics a shift from slow- to fast life history as the environment becomes more favorable for wild pigs.

Probability of establishment increased with propagule size in all environments, but stabilized in environments with a mast community and with agricultural subsidy (Fig. 2). In each of these environments, a propagule size ≥50 led to an establishment probability of >90%. While establishment probability in the environment with one mast species did not exceed 20% by a propagule size of 100, when we increased propagule size to 10,000 to obtain an estimate of the maximum establishment probability in this environment, establishment probability stabilized at 26% once propagule size reached 2,000 individuals (see Supplementary Note S1). We found that there was an effect of initial age structure on *λ*_s_, but the relationship depended on environment (Fig. 3). In the environment with one mast species, *λ*_s_ was highest when the number of juveniles in the propagule was low (Fig. 3a). In contrast, in environments with a mast community and agricultural subsidy, *λ*_s_ was much higher overall and it was highest when the number of juveniles was greater (Fig. 3b,c). *λ*_s_ calculated from long-term projections of the PPM (projecting populations under a single quality of mast for 10,000 years) were similar to previous estimates from the literature that calculated *λ* using the dominant eigenvalue^25^ for the intermediate- and good mast qualities (Table 1). However, in the poor mast quality, our long-term projection approach yielded a higher *λ*_s_ compared to the dominant eigenvalue calculation of Bieber and Ruff^25^.

**Table 1:** Comparing long-term estimates of stochastic growth rate (*λ*_s_) in wild pigs with those from the eigenanalysis (*λ*) in the literature^25^.

Mast quality	Estimated *λ* from eigenanalysis	Median *λ*_s_ estimate	95% CrI of *λ*_s_
poor	0.85	0.98	0.96–1.02
intermediate	1.09	1.10	1.06–1.14
good	1.63	1.61	1.57–1.64

## Discussion

When biologists use population dynamic modeling for conservation and management, it is useful to consider the timescale of interest^66^. For many such applications we are interested in the near-term population dynamics following a natural perturbation (e.g., fire or flood), a management intervention (e.g., reintroduction of a locally extirpated threatened species or culling of a problematic invasive species), or the novel introduction of an invasive species^50,57^. In these cases, populations will often not be at equilibrium (i.e., age structure may be different from stable age structure), causing populations to violate an important assumption of asymptotic population projections and to experience transient population dynamics^56,61^.

It is common for inference about population dynamics to use long-term projections and evaluate the asymptotic state of a population after the transient period^25,67^. These types of models can be useful for populations that quickly achieve asymptotic state. For example, our results using transient models to simulate wild pig populations under constant good and intermediate forage conditions were very similar to estimates of growth rate from asymptotic analyses (using the largest eigenvalue^25^; Table 1). However, long-term models that do not consider transient effects might be inaccurate for non-stable populations, as transient effects could persist for many generations^54,56,68,69^. This discrepancy is evident in the difference between the transient and asymptotic long-term growth rate predictions of wild pigs in a constantly poor habitat (Table 1). Short-term transient dynamic models can be more applicable to populations in a variety of situations. For newly introduced species of management concern, we are often more interested in the few years following release into a new environment, when the assumptions of asymptotic population dynamics do not necessarily apply^50^. Even for species that reside in the same location for long periods of time there are external effects on ecological systems (e.g., climatic oscillations, major environmental disturbances, and even geological changes) that might alter the age structure and the resulting population growth rate, making models that explicitly investigate short-term transient dynamics more relevant^66^.

Bieber & Ruf evaluated the elasticity of population growth rate to changes in wild pig vital rates using long-term simulations and proposed that wild pigs shift between ends of the slow-fast continuum of life history strategies^25^. They found that in low forage conditions, elasticity of growth rate to adult survival was highest, but elasticity of growth rate to juvenile survival was the highest in high forage conditions. Based on these results they proposed that wild pigs utilize a slow life history in poor environments and shift to a fast life history in good environments. We used a different approach to build upon their work. Instead of evaluating elasticities of growth rate to the proportion of good and poor mast years, we simulated different types of environments to which wild pigs might be exposed (with different amounts of forage) and varied the initial demographic distribution of the population to evaluate how these factors would affect growth rate. Our results suggest that in an environment with low amounts of forage, adults contribute more to population growth (Fig. 3a), while in environments with more resources, juveniles are more valuable for growth (Fig. 3b,c). This shift in growth strategy mimics a shift from a slow to a fast life history, but since we did not observe an evolutionary change, we cannot conclude that wild pigs utilized different life histories in different types of environments. Specifically, our model did not account for natural selection. Nevertheless, it appears that when forage is abundant, wild pigs are able to take advantage of both high fecundity and high survival: they produce many offspring with a high probability of survival. Wild pigs’ capability of coupling high fecundity and high survival in good forage conditions is one potential explanation for their rapid expansion in their invasive range^70^.

Our model predicted that environments with more forage would result in wild pig populations that grow rapidly (Fig. 1) and have a high probability of establishment following introduction (Fig. 2). We would expect that in these environments where wild pigs have greater access to forage, management should occur quickly following introduction in order to reduce the probability of establishment. Additionally, the frequency of mast in the future is predicted to increase in many locations due to climate change^71^, which might lead to faster growth rates and greater establishment success once wild pigs are introduced into new environments that have mast trees. The potential for more available forage in the future enforces the value of increased focus on preventing novel introductions and escape of wild pigs.

In the simulated environment with one mast species, our models suggested a declining or stable population size (median *λ*_s_ < 1, Fig. 1a) and low establishment probability (Fig. 2). When we increased propagule size to dramatically large numbers (up to 10,000) in this environment, probability of establishment did not exceed 26% (see Supplementary Note S1). It is possible that in low mast environments, populations are sustained either by dietary subsidy, switching their diets to other food types (as they do in locations lacking mast trees^27–30,32,40^), or by larger propagule numbers (multiple instances of wild pig introduction). It is also possible that vital rates for wild pigs in many locations are different from those found in Germany that were used in this study. Further research into wild pig populations in low forage environments might help explain their ability to persist despite relatively poor resources.

Our approach has several limitations that need to be addressed. Our simulations only account for a propagule number (i.e., number of events when pigs are released into a new area) of one. In reality, wild pigs are probably introduced into the same location multiple times^21^, which could lead to rescue effects (both demographic and genetic) for small populations on the brink of extinction^72^, and increase their probability of establishment. Future implementations of our approach could experiment with increased propagule number. Additionally, our matrix model approach does not account for non-linearity in population growth (e.g., density dependence). We also made the assumption that all animals in a population experienced the same probability of survival and reproduction at each time step; specifically, we did not implement an individual-based model such as the Gillespie algorithm^73^. However, we ran simulations where vital rates were drawn from a random gamma distribution, which led to increased variability in growth rate, but did not affect inference (see Supplementary Note S2). Furthermore, the vital rate data used in this study were collected in Germany, and extending our results into the invasive range assumes that vital rates are the same. It is possible that vital rates could be different in different locations (and that forage quality might affect vital rates differently), but we used these data as they were the only ones available for wild pigs under different forage conditions.

Additionally our approach could be extended by including population management practices such as culling, increased hunting, or fertility controls at different stages following wild pig introduction. When a new population of wild pigs is discovered, managers often attempt to eradicate this population quickly, before it establishes and spreads^74^. Future utilizations of this method can evaluate the efficacy of different management plans. For example, if a removal technique reduces adult survival or a land management strategy that inhibits wild pig access to agriculture reduces fertility, these parameters can be modified in models and their effect on population growth estimated. One of us (RSM) addresses these types of questions elsewhere^75^. Our method could also be extended by projecting the probability of growth and establishment of wild pig populations based on mast and agriculture. Currently, little geo-spatial data exist on the global distribution and abundance of mast tree species, but we are working on developing such data for North America and Europe (*Unpublished data*). Incorporating these spatial data with the models presented here will hopefully allow us to project the establishment potential for wild pigs in many locations in the Northern Hemisphere. This will help us evaluate the risk associated with wild pig release or escape into new locations.

We illustrated the use of an approach that has various applications including predicting the dynamics of populations introduced into new areas. Our approach allowed us to vary the propagule age structure, which can be a valuable tool for conservation and management planning. When planning reintroduction of threatened and endangered species for conservation, varying the propagule age structure can allow managers to choose the optimal age structure that is most conducive to population growth. For invasive species, managers often do not know the age structure of the propagule that arrives in a new location, and it is useful to consider all possibilities when projecting growth and establishment probability. This approach can also be used to predict the dynamics of species whose vital rates are dependent on environmental conditions that fluctuate (e.g., pulsed resource consumers and other species strongly dependent on a resource), as fluctuating population sizes will prevent stable population dynamics from arising^43,76^; the dynamics of populations responding to perturbations; and the dynamics of species whose ranges are expanding naturally. In these situations, populations will not be at stable conditions, so we argue that researchers should include transient dynamics in models.

## Methods

### Biological data for simulations

Researchers of wild pig biology have previously combined and synthesized estimates of survival and fecundity in part of their native range (Germany) for each age class in three types of years (i.e., poor, intermediate, and good) that are distinguished by mast quality^25,77–79^. These studies measured mean survival and fecundity for three age classes: juvenile (0-1 year), sub-adult (1-2 years), and adult (>2 years), but unfortunately, they did not report variances associated with these estimates. Bieber and Ruf used these vital rate estimates to create population projection matrices (PPMs) for wild pigs in each type of year quality (**A**_poor_, **A**_intermediate_, and **A**_good_; see Supplementary Table S3) to estimate the influence of forage quality on asymptotic population growth^25^. We used these PPMs with vital rate data from Germany for simulations because this is the only location (that we are aware of) from which data have been collected in each of the three types of forage conditions.

We simulated three types of environments to which wild pigs might be exposed in their native and invasive habitats. The vital rate data were collected in areas where some forests contain only one species of masting tree, the common beech (*Fagus sylvatica*^80^). A record of mast for this species over 114 years from Germany from 1800-1962^81^ showed that *F. sylvatica* had good masts (>100 full nuts per tree/7 minute sample) in 17.5% of the years, intermediate masts (51–100 full nuts per tree/7 minute sample) in 17.5% of the years, and poor masts (<51 full nuts per tree/7 minute sample) in 65% of the years. Therefore, we used these proportions of the three types of mast years for the set of simulations for *one mast tree species*. Few records exist for nut production from an entire mast community, but one such study exists for an oak (*Quercus* spp.) forest at Hastings Reservation, California, USA, where researchers collected data over 12 years^82^. In this community that contained five oak species (*Q. lobata*, *Q. douglasii*, *Q. chrysolepis*, *Q. agrifolia*, and *Q. kelloggii*), a good mast year occurred for at least one species in 33% of years, an intermediate year occurred for at least one species (without a good year occurring for any species) in 50% of years, and all species had poor mast in 17% of years. We used these proportions of the three types of mast years for the set of simulations where wild pigs are exposed to a *mast community*. Since agriculture has been associated with high reproductive success in wild pigs^83^, in an environment where their diet includes agricultural subsidy (i.e., they are able to easily consume excessive quantities of protein- and fat- rich crops regardless of mast quality), their vital rates might respond similarly to a good mast year^41,84^. Therefore, we also ran a set of simulations where 100% of years were good mast years to resemble an environment with *agricultural subsidy*. Some studies have shown that there may be negative autocorrelation among mast qualities (e.g., a poor mast year is likely to follow a good mast year^85^), while others have shown that it is more difficult to establish a pattern in mast and that good mast years can occur sequentially^82^. Therefore, in each type of simulated environment, we allowed the sequence of years to vary randomly (except in *agricultural subsidy*, where year quality did not vary). Bieber & Ruf used the same vital rate data to simulate environmental conditions to which wild pigs might be exposed in their native range^25^. Using mast records from beech in northern Germany they created a time series of the three different mast qualities and simulated populations for 10,000 years. For comparison, we used the same method of creating a time series of mast qualities and simulated populations using our transient dynamic method for 10^6^ realizations of both 10 years and 10,000 years (see Supplementary Note S4).

### Simulation procedure

For each of the three environments described, we used female-only PPMs to simulate population size over 10 years following a propagule’s introduction into a new environment. All modeling and simulations were conducted using custom scripts in R^86^; functions for implementing all simulations are provided as supporting information (see Supplementary Methods S5).

The demographic distribution in each subsequent year (**n**_**t+1**_) was estimated as:

$${{\boldsymbol{n}}}_{{\boldsymbol{t}}+{\bf{1}}}={\Vert {\hat{{\boldsymbol{A}}}}_{{\boldsymbol{t}}}{\hat{{\bf{n}}}}_{{\boldsymbol{t}}}\Vert }_{1}\ast {{\boldsymbol{n}}}_{{\boldsymbol{t}}}\ast {{\boldsymbol{A}}}_{{\boldsymbol{t}}}$$

where **A**_**t**_ is the PPM specific to the year quality for the simulated year, $\hat{{\bf{A}}}$_**t**_ is the standardized PPM (calculated by dividing **A**_**t**_ by *λ*_max_, the dominant eigenvector of **A**_**t**_), ${\hat{{\bf{n}}}}_{{\boldsymbol{t}}}$ is the standardized demographic distribution (i.e., the proportion of individuals in each age class) at the current time step, ${\Vert {\hat{{\boldsymbol{A}}}}_{{\boldsymbol{t}}}{\hat{{\bf{n}}}}_{{\boldsymbol{t}}}\Vert }_{1}$ represents the 1-norm of the product of ${\hat{{\boldsymbol{A}}}}_{{\boldsymbol{t}}}{\hat{{\bf{n}}}}_{{\boldsymbol{t}}}$ and is equal to the reactivity^52,54,59,87^.

For each simulated environment, 10^6^ realizations were used for each set of initial population sizes (after preliminary finding that results did not change with more realizations). Propagule sizes of 1, 2, 5, 10, 20, 30, 40, 50, 60, 70, 80, 90, and 100 were used as the number of females introduced in year one. We used these propagule sizes because this is a range of individuals that could potentially be introduced either accidentally (i.e., escape from a pig farm or truck), or intentionally to establish a recreational hunting population^9,23^. In each realization, the individuals in the propagule were randomly assigned to age classes (juvenile, sub-adult, and adult) using Latin hypercube sampling^88^. In subsequent generations, we assumed a sex ratio of 1:1 for litters. In the environments with one mast species and a mast community, the sequence of mast quality varied randomly for each realization (in the agriculturally subsidized environment, mast quality was treated as good in every year, so it did not vary). Simulations ran for 10 iterations (years) post introduction.

For each realization, the stochastic population growth rate (*λ*_s_) was calculated across the 10 years of the sequence. We used a regression-based approach to incorporate population size from all years in estimates of *λ*_s_. The first step in calculating *λ*_s_ was to calculate the intrinsic rate of population growth (*r*)^89^ as the slope of the regression between the logarithm of simulated population size and time (in years), then *λ*_s_ was calculated as: *λ*_s_ = e^*r*^, following the example of other researchers^90,91^. For each environment, we calculated probability of establishment based on two conditions: if growth rate was positive (*λ*_s_ > 1), and the population size after 10 years of simulation was at least 60 individuals, then the population in that realization was considered established. We used 60 individuals as the threshold for establishment because this is the number of individuals where *λ*_s_ stabilized in all simulated environments (described in Results).

Ecologists often evaluate population growth rate by simulating populations for long periods of time (e.g., 10,000 years) and evaluating dynamics^43,92^. To compare how transient dynamics may influence estimates of age class contributions to population growth versus longer term evaluations, we conducted longer simulations as well. Specifically, long-term transient population growth rate was calculated over 10,000 years for each type of mast year by holding environmental quality constant, using a starting population size of 60 individuals. We used 10,000 realizations (each for 10,000 years) for each of the three types of mast years.

### Data availability

Empirical data used for simulations were previously published^25,93^ and are reproduced in Supplementary Table S3.

## Electronic supplementary material

Supplementary Material
