# Supplementary material for: Propagule size and structure, life history, and environmental conditions affect establishment success of an invasive species
Source: Sci Rep. 2018 Jul 9;8:10313. doi: 10.1038/s41598-018-28654-w (PMC6037743; doi:10.1038/s41598-018-28654-w)
Supplement: Supplementary file 1 — Supplementary Material [file 41598_2018_28654_MOESM1_ESM.pdf]

**Propagule size and structure, life history, and environmental conditions affect establishment success of an invasive species**

Michael A. Tabak, Colleen T. Webb, Ryan S. Miller

**Propagule size and structure, life history, and environmental conditions affect establishment success of an invasive species**

Michael A. Tabak, Colleen T. Webb, Ryan S. Miller

**Supplementary Note S1: Simulation results from increasing propagule size in the *one mast tree* environment.**

For the environment with one mast species, probability of establishment did not exceed 20% and it did not stabilize with increasing propagule size under the conditions examined in the manuscript (a maximum propagule size of 100 individuals). To determine the stabilization point and the maximum establishment probability in this type of simulated environment, we ran simulations with larger propagule sizes, up to 10,000 individuals. The probability of establishment stopped increasing and stabilized beyond a propagule size of 2,000 individuals. The maximum establishment probability that we observed was 26.3%.

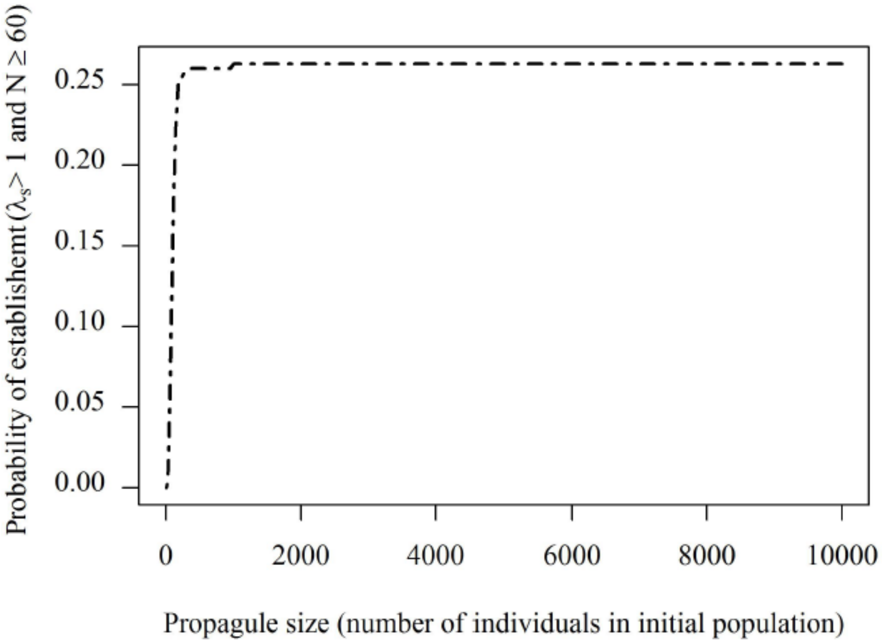

**Propagule size and structure, life history, and environmental conditions affect establishment success of an invasive species**

Michael A. Tabak, Colleen T. Webb, Ryan S. Miller

**Supplementary Note S2: Simulations in which vital rates were drawn from a random gamma distribution.**

In addition to those simulations described in the manuscript, we conducted simulations in which each vital rate of the population projection matrix was drawn randomly from a gamma distribution where the mean of the distribution was the mean vital rate reported in Appendix S1 and the variance of the distribution was 20% of the mean. Readers can experiment with these types of simulations and with different proportional variances using the code in Appendix S5 and setting stoch_A = TRUE.

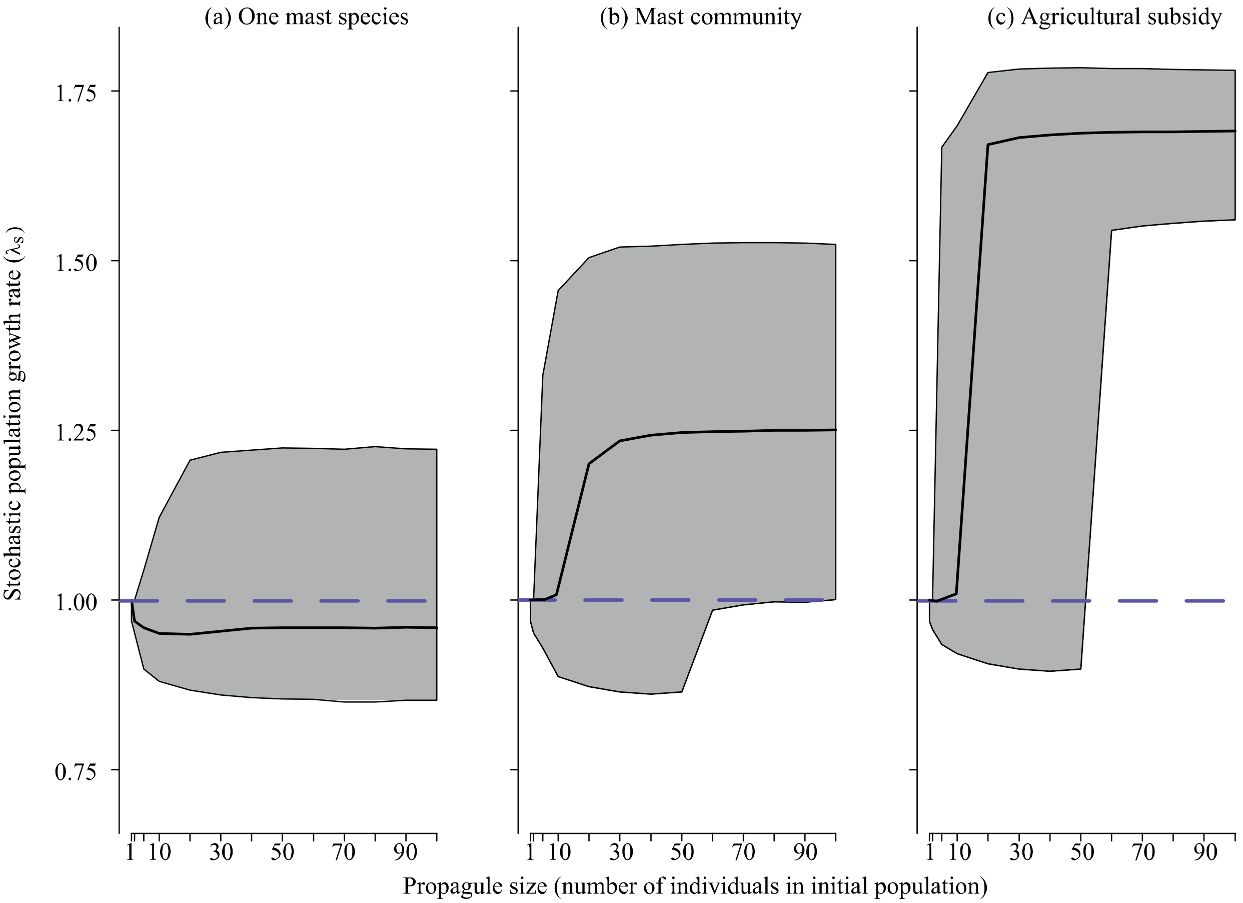


Stochastic population growth rate ($\lambda_s$) in simulations with stochastic vital rates was very similar to that with deterministic vital rates as seen in Fig. 1 of the main text, except that we found increased variability (and larger credible intervals) in the stochastic projections.

Nevertheless, the probability of establishment was largely unaffected by allowing the vital rates to be stochastic (compare with Fig. 2 in the main text).

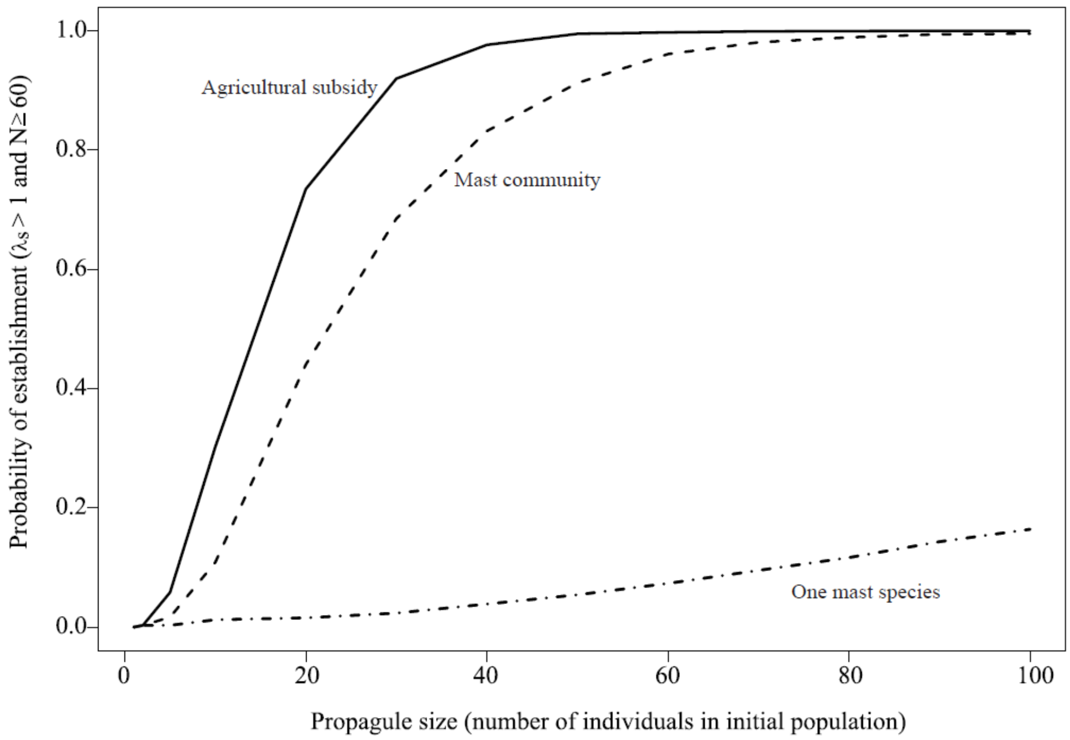


Therefore, we only present and discuss the deterministic simulation in the manuscript.

**Propagule size and structure, life history, and environmental conditions affect establishment success of an invasive species**

Michael A. Tabak, Colleen T. Webb, Ryan S. Miller

## Supplementary Table S3: Table of vital rate data used for analysis in this paper

Table 1: Vital rate estimates for wild pigs from multi-year studies under different environmental conditions[1]. Fertility is defined as the number of juveniles at time $t + 1$, per individual in this age class at time $t$[2].

| Mast Quality | Age class | Mean litter size | Proportion reproducing | Fertility | Survival rate |
|---|---|---|---|---|---|
| Poor | Juvenile | 3.5 | 0.30 | 0.13 | 0.25 |
| | Sub-adult | 4.5 | 0.80 | 0.56 | 0.31 |
| | Adult | 6.3 | 0.90 | 1.64 | 0.58 |
| Intermediate | Juvenile | 4.0 | 0.40 | 0.26 | 0.33 |
| | Sub-adult | 5.5 | 0.85 | 0.94 | 0.40 |
| | Adult | 6.5 | 0.90 | 1.93 | 0.66 |
| Good | Juvenile | 4.5 | 0.50 | 0.59 | 0.52 |
| | Sub-adult | 6.5 | 0.90 | 1.76 | 0.60 |
| | Adult | 6.8 | 0.95 | 2.29 | 0.71 |

Note: A version of this table was previously published[1], but this table provides the corrected information[3].

## References

1. Bieber, C. & Ruf, T. Population dynamics in wild boar *Sus scrofa*: ecology, elasticity of growth rate and implications for the management of pulsed resource consumers. *J. Appl. Ecol.* **42,** 1203–1213 (2005).

2. Caswell, H. *Matrix Population Models: Construction, Analysis, and Interpretation*. (Sinauer associates, Inc., 2001).

3. Bieber, C. & Ruf, T. Erratum: Population dynamics in wild boar *Sus scrofa*: ecology, elasticity of growth rate and implications for the management of pulsed resource consumers. (2005).

**Propagule size and structure, life history, and environmental conditions affect establishment success of an invasive species**

Michael A. Tabak, Colleen T. Webb, Ryan S. Miller

## Supplementary Note S4: Simulation results using the method of creating time series used by Bieber & Ruf[1]

### Simulation and calculation of $\lambda_s$

We ran our set of simulations using the system for assigning mast quality in a time series that was outlined in Bieber and Ruf[1]. First, good mast years were included in the sequence at intervals drawn from a Poisson distribution with a mean of 7. Then, each year following a good mast year was assigned as a poor mast year. The remaining years in the sequence were randomly assigned to either poor or intermediate quality mast years, while ensuring that a proportion of 34% poor mast years was present in the entire time series. We varied propagule size as described in the text and used $10^6$ realizations for each propagule size. We ran this simulation for 10,000 years, as was conducted by Bieber and Ruf, and we also ran for 10 years, to compare with our other simulations.

Bieber and Ruf found $\lambda_s$ = 1.05 for their 10,000 year simulation[1]. When we used 10 years of simulation, the stochastic population growth rate ($\lambda_s$) was similar to our results from an environment with a mast community (Fig 1b in main text). With a propagule size > 10, median $\lambda_s$ was higher than the value found by Bieber and Ruf[1]. When we used 10,000 years of simulation, the credible interval was very narrow and $\lambda_s$ was close to one, but the median value found by Bieber and Ruf was not included in the credible interval that we observed from our simulations.

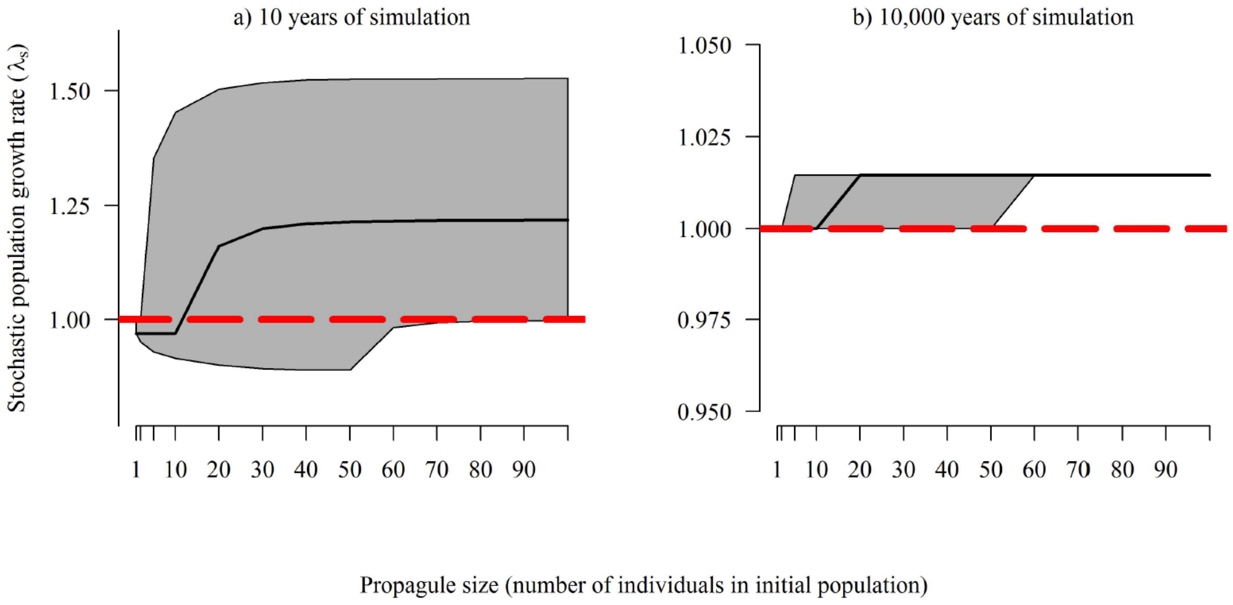

Population growth rate ($\lambda_s$) was much lower in the 10,000-year simulation than in the 10-year simulation. It is likely that over such a long time period, the transient effects that were observed in the short time period no longer have a strong influence on population dynamics. In the main text, we discuss the relative relevance of different lengths of simulation.

## Probability of establishment

We also calculated probability of establishment (the number of realizations with $\lambda_s > 1$ and population size $\geq 60$ in the final year of simulation) in the same manner as described in the text. For the 10 year simulation, the probability of establishment using this sequence of years was similar to that of a mast community (Fig 2 in main text). When we simulated for 10,000 years, the probability of establishment was higher.

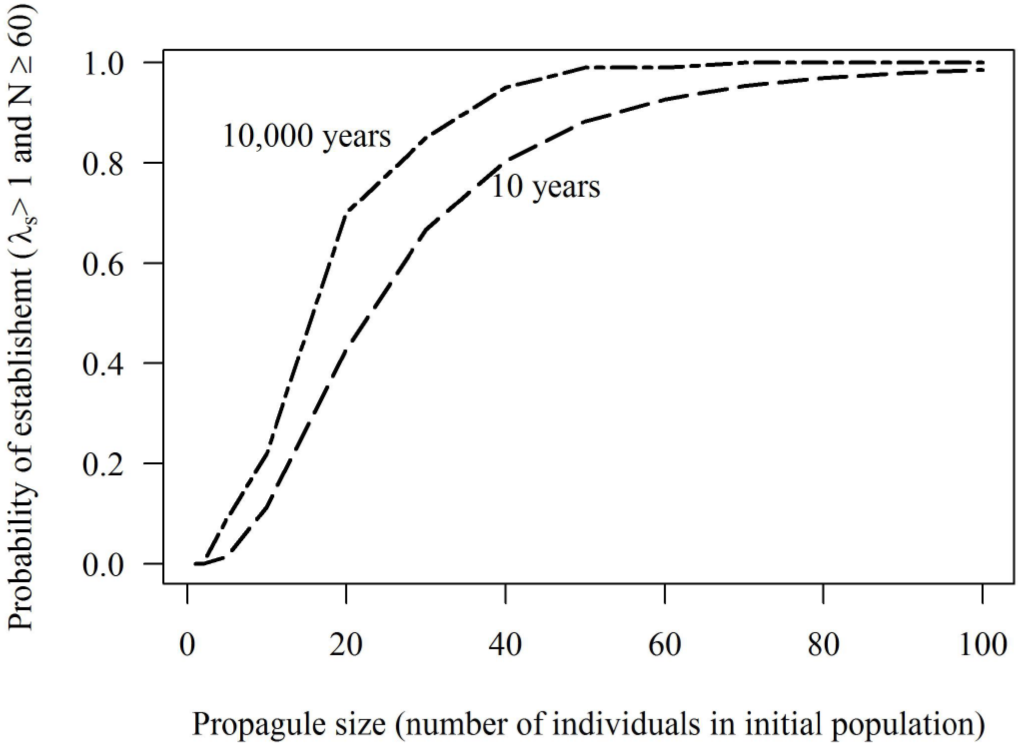


The discrepancy in establishment probability in the different length simulations is likely a result of the fact that in a longer simulation, more populations are likely to reach the threshold population size of 60 individuals (and thus be classified as established) by the final step in the time series.

## References

1. Bieber, C. & Ruf, T. Population dynamics in wild boar *Sus scrofa*: ecology, elasticity of growth rate and implications for the management of pulsed resource consumers. *J. Appl. Ecol.* **42,** 1203–1213 (2005).

**Propagule size and structure, life history, and environmental conditions affect establishment success of an invasive species**

Michael A. Tabak, Colleen T. Webb, Ryan S. Miller

**Supplementary Methods S5: Computer code (R) for conducting the simulations described in methods.**

```
## Propagule size and structure, life history, and environmental conditions
## affect establishment success of an invasive species
## Michael A. Tabak, Colleen T. Webb, Ryan S. Miller

## Supplementary Methods S5: Computer code (R) for conducting the simulations described in methods.

# Note to user: if you are using this code for species other species (other than
# sus scrofa), you will need to change the transition matrices that are specified
# in the code below. These are denoted as A.p, A.i, and A.g for poor,
# intermediate, and good year transition matrices.

# This code requires one package. You need to run install.packages("lhs") if you don't already have it
require(lhs)

# bounds function (1 timestep)
bounds <- function (
  #   this function calculates "reactivity" according to Townley et al. 2007 (J Ap Ecol)
  #   or it calculates the combination of 'reactivity' and 'first time step attenuation' according
  #   to Stott et al 2011 (Ecol Lett) and 2012 (methods ecol evol)
  A,
  vector = "n",
  return.N = TRUE,
  std.dev=0.2,
  stoch_A=FALSE,
  med.proj=FALSE,
```

```
  prop.var=TRUE, # variance is a proportion of matrix element
  var.prop=0.2 # proportion of matrix element that variance is
)

{

  order <- dim(A)[1]
  #- make A matrix stochastic
  if(stoch_A){
    # set variance for stochastic matrix
    var <- matrix(0, order, order)
    if(prop.var){
      for(i in 1:order){
        for(j in 1:order){
          var[i,j] = A[i,j]*var.prop
        }
      }
    } else {
      var = matrix(rep(std.dev, (order*2)), order, order)
    }

    A.stoch <- matrix(NA, order, order)
    for(i in 1:order){
      for(j in 1:order){
        if(A[i,j]==0){
          A.stoch[i,j] = 0
        } else{
          mu = A[i,j]
          a <- mu^2/var[i,j]
          s <- var[i,j]/mu
          A.stoch[i,j] = rgamma(1, shape=a, scale=s)
        }
```

```
      }
    }
  } else{
    # when not using a stochastic matrix.
    # This is the deterministic version of model used in the paper
    A.stoch <- A
  }

  if(sum(A.stoch)==0){
    # take care of matrices where all vital rates are simulated to be 0
    return(list(reactivity=0, N=0))
  } else {
    M <- A.stoch
    eigvals <- eigen(M)$values # take eigenvalues of matrix A
    lmax <- which.max(Re(eigvals)) # find the maximum eigenvalue of matrix A. Re() turns eigenvalues into numbers
    lambda <- Re(eigvals[lmax]) # take the largest eigenvalue (and turn into real number) = lambda
    Ahat <- M/lambda

    n0 <- vector
    vector <- n0/sum(n0) # turns into proportion in each age class instead of total numbers
    reactivity <- sum(Ahat %*% vector) # reactivity
    if (sum(n0) <= 0) {
      return(list(bounds = reactivity, N=0))
    } else {
      if (return.N) {

        # this switch allows us to change between median projection and reactivity
        if(med.proj){
          Nt <- A.stoch %*% n0 # median / project
        } else{
          Nt <- (A.stoch * reactivity) %*% n0  # bounds / reactivity
```

```
        }

        return(list(reactivity = reactivity, N = Nt))
      }
      else {
        return(reactivity)
      }
    }
  }
} # end bounds () function


round_lh <- function(x) {
  # rounding function to ensure that initial population size is an integer
  y <- floor(x)
  indices <- tail(order(x-y), round(sum(x)) - sum(y))
  y[indices] <- y[indices] + 1
  return(y)
}

#- take bounds function over multiple timesteps
boundsTimesteps <- function (
  # This function simulates population size over timesteps.
  # This function assumes that the stage structure
  # at a given time is based on the equilibrium stage structure at the
  # previous time. It uses the bounds function, which is the maxium step
  # (amplification or attenuation) for one timestep.

  initpop = 3,                  # initial population size
  timesteps = 10,               # number of timesteps
  qualityMethod = "stoch",      # how we set up year qualities. deter (determinstic) uses a specific sequence
  # stoch (stochastic) uses a random sequence based on proportion of year qualities
```

```
  yearSeq = c("good", "poor", rep("intermed", 6), "good", "poor"), # deterministic sequence of years
  p.g = 0.4,                    # probability of good years
  p.i = 0.4,                    # probability of intermeidate years
  p.p = 0.2,                    # probability of poor years
  std.dev = 0.2,                # sd of matrix elements
  preStepsQual = "good",        # value for year before timesteps c("good", "intermd", "poor"),
  initpop.type = "vec",         # is initial population size a vector or scalar
  med.proj = FALSE,             # calcuting reactivity if FALSE; median if TRUE
  verbose=FALSE,                # have smaller output when using as part of function
  prop.var=TRUE,                # variance is a proportion of matrix element
  var.prop=0.2,                 # proportion of matrix element that variance is
  makePlot = FALSE,             # have function output a plot?
  latinHS = FALSE               # latin hypercube sampling? if FALSE, random sampling for initial distribution
){

  require(lhs)


  #- ppms
  # poor
  A.p <- matrix(c(0.13,0.56,1.64,
                  0.25,0,0,
                  0,0.31,0.58), nrow=3, ncol=3, byrow=TRUE)


  # intermediate
  A.i <- matrix(c(0.26,0.94,1.93,
                  0.33,0,0,
                  0,0.40,0.66), nrow=3, ncol=3, byrow=TRUE)


  # good
  A.g <- matrix(c(0.59,1.76,2.29,
                  0.52,0,0,
                  0,0.60,0.71), nrow=3, ncol=3, byrow=TRUE)
```

```r
order <- ncol(A.g)

#- create a distribution of good, intermediate, and poor years
# starting year quality
startA <- NA
if (preStepsQual == "good"){
  startA = A.g
} else if (preStepsQual == "intermed"){
  startA = A.i
} else if (preStepsQual == "poor"){
  startA = A.p
}

#- timesteps quality
q.samples <- c(rep("good", timesteps*p.g), rep("intermed", timesteps*p.i),
               rep.int("poor", times=timesteps*p.p))

if (qualityMethod == "deter"){
  q.temp = yearSeq
} else {
  q.temp = sample(q.samples, size=timesteps, replace=TRUE) #sample(q.samples, replace=FALSE)
}

q.list <- list()
for (i in 1:timesteps){
  if (q.temp[i] == "good"){
    q.list[[i]] = A.g
  } else if (q.temp[i] == "intermed"){
    q.list[[i]] = A.i
  } else if (q.temp[i] == "poor"){
    q.list[[i]] = A.p
  }
```

```
}
quality <- c(preStepsQual, q.temp)


#- start analyzing
N.years <- matrix(0, ncol=3, nrow=(timesteps+1))
if (initpop.type=="vec"){ # when sending an initial pop vector from the next function
  N.years[1,] = initpop
  for (i in 1:timesteps){
    N.years[(i+1),] = bounds(q.list[[i]],
                             vector=N.years[i,],
                             return.N=TRUE,
                             std.dev=std.dev,
                             prop.var=prop.var, var.prop=var.prop,
                             med.proj=med.proj)$N
  }
} else {
  # set initial population distribution because not specified as function input
  # if an initial population distribution is not supplied, randomly assign
  # individuals to age classes.
  if(latinHS == TRUE){
    hypercubes <- randomLHS(1, order)
    perms <- round(hypercubes/rowSums(hypercubes), digits=2)
    age.dist <- round_lh(perms*initpop)
  } else {
    pop.vector <- seq(1,order)
    classes <- sample(pop.vector, initpop, replace=TRUE)
    age.dist <- rep(NA, order)
    for(j in 1:order){
      age.dist[j] = sum(classes==j)
    }
  }
```

```
  N.years[1,] <- age.dist # randomly assigned age class for timestep 1

  # now run simulation for subsequent timesteps
  for (i in 1:timesteps){
    N.years[is.na(N.years)] <- 0
    if (sum(N.years[i,] <= 0)){
      N.years[(i+1),]=0
    } else {
      N.years[(i+1),] = bounds(q.list[[i]],
                               vector=N.years[i,],
                               return.N=TRUE,
                               std.dev=std.dev,
                               prop.var=prop.var, var.prop=var.prop,
                               med.proj=med.proj)$N
    }
  }
}
# calculate timestep specific lambda
lambda <- rep(0, timesteps+1)
for (i in 2:(timesteps+1)){
  lambda[i] = sum(N.years[i,])/sum(N.years[(i-1),])
}

# put the data together
if (verbose){
  out <- data.frame(quality, N.years, lambda)
} else {
  if (initpop.type=="vec") {
    out <- list("N.years"=N.years,
                "lambda"=lambda)
  } else{
    out <- list("N.years"=N.years,
```

```
                "lambda"=lambda,
                'init.age.dist'=age.dist)
    }
  }
  #return(out)

  # make figure
  if (makePlot){
    p.plot <- plot(1:(timesteps+1), rowSums(out$N.years), type='l', ylim=c(0, (max(out$N.years)+10)),
                ylab="population size", xlab="time step",
                main=c("p.good=", paste(p.g), 'p.intermed=', paste(p.i), 'p.poor=', paste(p.p)))
    return(list(p.plot, out))
  } else {
    return(out)
  }

} # end transient steps function

#- function to run simulations
simStepsBounds <- function (
  # this function runs multiple simulations of populations over timesteps.
  # It calls the function transientSteps().

  iter = 100,                    # number of simulations to run. must be greater than 1
  initpop = 10,                  # initial population size
  timesteps = 10,                        # number of timesteps
  preStepsQual = "poor",        # value for year before timesteps c("good", "intermd", "poor"),
  qualityMethod = "stoch",      # how we set up year qualities. deter (determinstic) uses a specific sequence
  # stoch (stochastic) uses a random sequence based on proportion of year qualities
  yearSeq = c("good", "poor", rep("intermed", 6), "good", "poor"), # deterministic sequence of years
  p.g = 0.4,                     # probability of good years
  p.i = 0.4,                     # probability of intermeidate years
```

```
  p.p = 0.2,                    # probability of poor years
  std.dev = 0.2,                # sd of matrix elements
  initpop.type = "num",         # is initial population size a vector or scalar
  med.proj = FALSE,             # calcuting reactivity if FALSE; median if TRUE
  makePlot = FALSE,             # have function output a plot?
  plotCI = FALSE,               # plot 95% CI
  compareSSD = FALSE,           # compare simulated distribution to SSD
  prop.var=TRUE,                # variance is a proportion of matrix element
  var.prop=0.2,                 # proportion of matrix element that variance is
  est.thresh = 60,              # establishment threshold
  latinHS = TRUE
){

  #- timesteps quality # running this again so that I use the same sequence of years for each simulation
  #- ppms
  # poor
  A.p <- matrix(c(0.13,0.56,1.64,
                0.25,0,0,
                0,0.31,0.58), nrow=3, ncol=3, byrow=TRUE)

  # intermediate
  A.i <- matrix(c(0.26,0.94,1.93,
                0.33,0,0,
                0,0.40,0.66), nrow=3, ncol=3, byrow=TRUE)

  # good
  A.g <- matrix(c(0.59,1.76,2.29,
                0.52,0,0,
                0,0.60,0.71), nrow=3, ncol=3, byrow=TRUE)

  # order of matrix. Assuming all quality matrices have same number of age classes
  order <- dim(A.g)[1]
```

```
# set up for random
q.samples <- c(rep("good", timesteps*p.g), rep("intermed", timesteps*p.i),
               rep.int("poor", times=timesteps*p.p))

if (qualityMethod == "deter"){
  q.temp = yearSeq
} else { # if stochastic, sample from q.samples
  q.temp = sample(q.samples, size=timesteps, replace=TRUE) #sample(q.samples, replace=FALSE)
}

q.list <- list()
for (i in 1:timesteps){
  if (q.temp[i] == "good"){
    q.list[[i]] = A.g
  } else if (q.temp[i] == "intermed"){
    q.list[[i]] = A.i
  } else if (q.temp[i] == "poor"){
    q.list[[i]] = A.p
  }
}
quality <- c(preStepsQual, q.temp)

# run transientSteps multiple times
simmedN <- matrix(0, (timesteps+1), iter)
lambdas <- matrix(0, (timesteps+1), iter)
pop.vector <- seq(1,order)
age.dist <- rep(NA, order)
all.age.dist <- matrix(NA, nrow=iter, ncol=order)

simList <- list()
for (i in 1:iter){
```

```
  # randomly assign population distribution for first timestep
  if(latinHS == TRUE){
    hypercubes <- randomLHS(1, order)
    perms <- round(hypercubes/rowSums(hypercubes), digits=2)
    age.dist <- round_lh(perms*initpop)
  } else {
    pop.vector <- seq(1,order)
    classes <- sample(pop.vector, initpop, replace=TRUE)
    age.dist <- rep(NA, order)
    for(j in 1:order){
      age.dist[j] = sum(classes==j)
    }
  }

  startPop <- sum(age.dist)
  simList[[i]] = boundsTimesteps(initpop=age.dist, timesteps=timesteps,
                                 preStepsQual=preStepsQual,
                                 qualityMethod=qualityMethod,
                                 yearSeq=quality[2:(length(quality))],
                                 p.g=p.g, p.i=p.i, p.p=p.p, std.dev=std.dev,
                                 initpop.type=initpop.type, #'vec', # because this function is creating a vector
                                 med.proj=med.proj,
                                 prop.var=prop.var, var.prop=var.prop,
                                 makePlot=FALSE)
  simmedN[,i] = rowSums(simList[[i]]$N.years) # total simmed population size
  lambdas[,i] = simList[[i]]$lambda
  all.age.dist[i,] = age.dist
} # closes i loop

# calculate the stable stage distribution for each iteration at each timestep
ssd <- matrix(NA, nrow=iter, ncol=order)
ssdList <- list()
```

```
if(compareSSD){
  for(l in (1:timesteps)){
    for(i in 1:iter){
      #ssd[i,] <- stable.stage(q.list[[10]])*simmedN[10, i]
      ssd[i,] <- stable.stage(q.list[[l]])*simmedN[(l+1), i]
    }
    ssdList[[l]] = ssd
  }
} # closes compare SSD

# make plot of all simulations
if(makePlot==TRUE){
  p.plot <- plot(1:(timesteps+1), simmedN[,1], type='l', ylim=c(0, (max(simmedN)+10)),
               #p.plot <- plot(1:(timesteps+1), log10(simmedN[,1]+1), type='l', ylim=c(-1,
(max(log10(simmedN+1)))),
               xlab="timestep (year)", ylab="population size")
  for (i in 1:iter){
    p.plot <- lines(simmedN[,i])
  }
}

#- calculate stochastic lambda
lambda.s <- rep(NA, iter) # stochastic lambda, calculated following Zuniga-vega et al. 2007. Copeia
lambda.s2 <- rep(NA, iter) # calculated with log(N+1)
years <- 1:(timesteps+1)



for(i in 1:iter){
  # remove iterations where population size is too large
  if (max(simmedN[,i]) > 10^308) {
    lambda.s2[i] = 2 #NA
  } else {
```

```
    lambda.s2[i] <- exp(lm(log(simmedN[(1:(timesteps+1)),i]+1) ~ years)$coefficients[2])
    if (simmedN[(timesteps+1), i] > 0){
      lambda.s[i] <- exp(lm(log(simmedN[(1:(timesteps+1)),i]) ~ years)$coefficients[2])
    } else{
      lambda.s[i] = 0
    }
  }
}

#- calculate summary statistics
medianN <- rep(NA, (timesteps+1))
minN <- rep(NA, (timesteps+1))
maxN <- rep(NA, (timesteps+1))
medianLambda <- rep(NA, (timesteps+1))
minCI <- rep(NA, (timesteps+1))
maxCI <- rep(NA, (timesteps+1))

# credible interval
for(i in 1:(timesteps+1)){
  minCI[i] = quantile(simmedN[i,], 0.025, na.rm=TRUE)
  maxCI[i] = quantile(simmedN[i,], 0.975, na.rm=TRUE)
}
if(makePlot==TRUE & plotCI == TRUE){
  lines(1:(timesteps+1), minCI, type='l', xlab='time (years)', ylab='population size',
        ylim=c(0,(max(maxCI)+10)))
  lines(1:(timesteps+1), maxCI)
  polygon(c(1:(timesteps+1), rev(1:(timesteps+1))), c(maxCI, rev(minCI)),
          col="grey30")
}

establish <- mean(simmedN[(timesteps+1),] > est.thresh, na.rm=TRUE) # only looking at establishment in the 10th
year
```

```
estab2cond <- rep(NA, iter)
for(i in 1:iter){
  if(simmedN[(timesteps+1),i] > est.thresh && lambda.s2[i] > 1){
    estab2cond[i] <- 1
  } else{
    estab2cond[i] <- 0
  }
}

for (i in 1:(timesteps+1)){
  medianN[i] = median(simmedN[i,], na.rm=TRUE)
  minN[i] = min(simmedN[i,], na.rm=TRUE)
  maxN[i] = max(simmedN[i,], na.rm=TRUE)
  medianLambda[i] = median(lambdas[i,], na.rm=TRUE)
}
summaries <- data.frame(medianN, minN, maxN,
                        minCI, maxCI, # comment out this line and the above for loop to avoid using stan
                        medianLambda, quality)

if (makePlot==TRUE){
  return(list(p.plot, summaries, establish=establish, lambda.s=lambda.s))
  #return(list(simmedN,lambdas))
} else if (compareSSD){
  return(ssdList)
} else {
  return(list(summaries, estab2cond=estab2cond,
              establish=establish,
              lambda.s=lambda.s, lambda.s2=lambda.s2,
              all.age.dist=all.age.dist, simmedN=simmedN))
}
```

```
} # end simSteps function


#- function to simulate over different environmental conditions
boundsStepVaryingQualities <- function(
  initpop=15,
  iter=100,
  timesteps=10,
  std.dev = 0.2,                # sd of matrix elements
  est.thresh=60,
  prop.var=TRUE,                # variance is a proportion of matrix element
  var.prop=0.2,                 # proportion of matrix element that variance is
  nquals='some',                # if nquals==all -> projects seven different environments. Otherwise it projects only three
  med.proj = FALSE              # calcuting reactivity if FALSE; asymptotic if TRUE
){

  # run project timesteps function
  outlist <- list()
  n <- 7 # number of qualities
  n2 <- n+1
  prob.establish <- rep(NA, n) # add matrix to take lambda.s
  lambda.s <- matrix(NA, nrow=iter, ncol=n)
  lambda.s2 <- matrix(NA, nrow=iter, ncol=n)
  estab2cond <- rep(NA, n)

  if(nquals=='all'){
    for (i in 1:n){
     #         prob.establish[i] = simStepsBounds(initpop=initpop, qualityMethod="stoch",
     #                                   timesteps=10, p.g=(i/10), p.i=.2,
     #                                   p.p=((n2-i)/10),
     #                                   iter=iter,
     #                                   est.thresh=est.thresh,
```

```
    #                                  initpop.type="num",
    #                                  makePlot=FALSE)$establish
    outlist[[i]] <- simStepsBounds(initpop=initpop, qualityMethod="stoch",
                        timesteps=10, p.g=(i/10), p.i=.2,
                        p.p=((n2-i)/10), std.dev=std.dev,
                        iter=iter,
                        est.thresh=est.thresh,
                        med.proj=med.proj,
                        initpop.type="num",
                        prop.var=prop.var, var.prop=var.prop,
                        makePlot=FALSE)

    prob.establish[i] = outlist[[i]]$establish
    lambda.s[,i] = outlist[[i]]$lambda.s
    lambda.s2[,i] = outlist[[i]]$lambda.s2
    estab2cond[i] = mean(outlist[[i]]$estab2cond==1)
   }
   # make some properties of outlist
   prob.good <- seq(0.1, 0.7, by=0.1)
   df.out <- data.frame(prob.good, prob.establish)

  } else{
   #*** NOTE: this is where user needs to modify proportions of mast years if planning to change them
   outlist[[1]] <- simStepsBounds(initpop=initpop, qualityMethod="stoch",timesteps=10,
                        p.g=0.175, p.i=.175,p.p=0.65,
                        std.dev=std.dev,
iter=iter,est.thresh=est.thresh,med.proj=med.proj,initpop.type="num",prop.var=prop.var,
var.prop=var.prop,makePlot=FALSE)
   outlist[[2]] <- simStepsBounds(initpop=initpop, qualityMethod="stoch",timesteps=10,
                        p.g=1/3, p.i=1/2,p.p=1/6,
                        std.dev=std.dev,
iter=iter,est.thresh=est.thresh,med.proj=med.proj,initpop.type="num",prop.var=prop.var,
var.prop=var.prop,makePlot=FALSE)
```

```
    outlist[[3]] <- simStepsBounds(initpop=initpop, qualityMethod="stoch",timesteps=10,
                                  p.g=1, p.i=0,p.p=0,
                                  std.dev=std.dev,
iter=iter,est.thresh=est.thresh,med.proj=med.proj,initpop.type="num",prop.var=prop.var,
var.prop=var.prop,makePlot=FALSE)
    n <- 3
    prob.establish <- rep(NA, n)
    lambda.s <- matrix(NA, nrow=iter, ncol=n)
    lambda.s2 <- matrix(NA, nrow=iter, ncol=n)
    estab2cond <- rep(NA, n)
    for(i in 1:n){
      prob.establish[i] = outlist[[i]]$establish
      lambda.s[,i] = outlist[[i]]$lambda.s
      lambda.s2[,i] = outlist[[i]]$lambda.s2
      estab2cond[i] = mean(outlist[[i]]$estab2cond==1)
    }
    # make some properties of outlist
    environment <- c('low.mast', 'intermed.mast', 'high.mast')
    df.out <- data.frame(environment, prob.establish)
  }

  # make output list
  list.out <- list('prob.establish'=df.out,
                   'lambda.s'=lambda.s,
                   'lambda.s2'=lambda.s2,
                   'estab2cond'=estab2cond)
  return(list.out)
}

# test
#bs <- boundsStepVaryingQualities(iter=100)
```

```
#- write function to run for different initial conditions
diffInitialCond <- function(
  initialPops = c(2,5,10,20,30,40,50,60,70,80,90,100),
  iter=100,
  timesteps=10,
  std.dev = 0.2,               # sd of matrix elements
  est.thresh=60,
  prop.var=TRUE,               # variance is a proportion of matrix element
  var.prop=0.2,                # proportion of matrix element that variance is
  nquals='some',               # if nquals==all -> projects seven different environments. Otherwise it projects only three
  med.proj = FALSE             # calcuting reactivity if FALSE; median if TRUE
){

  # set up parameters
  if(nquals=='all'){
    prop.good <- prop.good <- seq(0.1,0.7, by=0.1) # probability of good years
  } else {
    prop.good <- c(0.1,0.3,0.7)
  }
  n <- length(prop.good)
  numSum <- 7 # number of summary stats
  simList <- list()
  summaryList <- list()
  summaryList2 <- list()
  proj.established <- matrix(NA, nrow=n, ncol=(max(initialPops)))#ncol=length(initialPops))
  prop.estab2cond <- matrix(NA, nrow=n, ncol=(max(initialPops)))#
  lam.s.sum <- matrix(NA, nrow=n, ncol=max(initialPops))
  X <- initialPops

  for (i in X){
    simList[[i]] = boundsStepVaryingQualities(initpop=i,
                                              iter=iter,
```

```
                                        timesteps=timesteps,
                                        std.dev=std.dev,
                                        est.thresh=est.thresh,
                                        prop.var=prop.var, var.prop=var.prop,
                                        med.proj=med.proj, nquals=nquals)
  proj.established[,i] = simList[[i]]$prob.establish[,2]
  prop.estab2cond[,i] = simList[[i]]$estab2cond
} # close i
#return(proj.established)}; proj.established <- diffInitialCond.bounds(iter=20)

for (j in 1:n){
  summaryList[[j]] <- matrix(NA, nrow=numSum, ncol=max(initialPops))
  summaryList2[[j]] <- matrix(NA, nrow=numSum, ncol=max(initialPops))

  for(k in X){
    summaryList[[j]][1,k] = mean(simList[[k]]$lambda.s[,j], na.rm=TRUE)
    summaryList[[j]][2,k] = median(simList[[k]]$lambda.s[,j], na.rm=TRUE)
    summaryList[[j]][3,k] = quantile(simList[[k]]$lambda.s[,j], 0.005, na.rm=TRUE) #0.025, na.rm=TRUE)
    summaryList[[j]][4,k] = quantile(simList[[k]]$lambda.s[,j], 0.995, na.rm=TRUE)#0.975, na.rm=TRUE)
    summaryList[[j]][5,k] = mean(simList[[k]]$lambda.s[,j] > 1, na.rm=TRUE)
    summaryList[[j]][6,k] = quantile(simList[[k]]$lambda.s[,j], 0.025, na.rm=TRUE) #0.025, na.rm=TRUE)
    summaryList[[j]][7,k] = quantile(simList[[k]]$lambda.s[,j],  0.975, na.rm=TRUE)#0.975, na.rm=TRUE
    #summaryList[[j]][6,k] = mean(simList[[k]]$estab2cond[j])
    # list for usting log(N+1)
    summaryList2[[j]][1,k] = mean(simList[[k]]$lambda.s2[,j], na.rm=TRUE)
    summaryList2[[j]][2,k] = median(simList[[k]]$lambda.s2[,j], na.rm=TRUE)
    summaryList2[[j]][3,k] = quantile(simList[[k]]$lambda.s2[,j], 0.005, na.rm=TRUE) #0.025, na.rm=TRUE)
    summaryList2[[j]][4,k] = quantile(simList[[k]]$lambda.s2[,j],  0.995, na.rm=TRUE)#0.975, na.rm=TRUE)
    summaryList2[[j]][5,k] = mean(simList[[k]]$lambda.s2[,j] > 1, na.rm=TRUE)
    summaryList2[[j]][6,k] = quantile(simList[[k]]$lambda.s2[,j], 0.025, na.rm=TRUE) #0.025, na.rm=TRUE)
    summaryList2[[j]][7,k] = quantile(simList[[k]]$lambda.s2[,j],  0.975, na.rm=TRUE)#0.975, na.rm=TRUE)
    #summaryList2[[j]][6,k] = simList[[k]]$estab2cond[j]
```

```
     #summaryList2[[j]][6,k] = mean(simList[[k]]$lambda.s2 > 1, na.rm=TRUE) # probability of growth across all initial population size values
   } # close k
  } # close j

  # calculate proportion of lambdas that are greater than 1 for each set of year qualities
  # only works with initial pops = c(2,5,10,20,30, 40, 50, 60); otherwise, need to edit this code
  more.one <- rep(NA, n)
  for(i in 1:n){
    more.one[i] = mean(c(simList[[2]]$lambda.s2[,i], simList[[5]]$lambda.s2[,i],
                         simList[[10]]$lambda.s2[,i], simList[[20]]$lambda.s2[,i],
                         simList[[30]]$lambda.s2[,i], simList[[40]]$lambda.s2[,i],
                         simList[[50]]$lambda.s2[,i], simList[[60]]$lambda.s2[,i]) >= 1)
  }

  proj.established2 <- proj.established[, colSums(is.na(proj.established)) != nrow(proj.established)]



  # remove null elements from list
  summaries.lam.s <- Filter(Negate(function(x) is.null(unlist(x))), summaryList)
  summaries.lam.s2 <- Filter(Negate(function(x) is.null(unlist(x))), summaryList2)

  # write a loop that will cycle though these lists and remove colSums(is.na())
  sumlam1 <- list()
  sumlam2 <- list()
  for(i in 1:n){
    sumlam1[[i]] <- summaries.lam.s[[i]][, colSums(is.na(summaries.lam.s[[i]])) != nrow(summaries.lam.s[[i]])]
    sumlam2[[i]] <- summaries.lam.s2[[i]][, colSums(is.na(summaries.lam.s2[[i]])) != nrow(summaries.lam.s2[[i]])]
  }
  #  return(sumlam1)};  diffInitialCond.bounds(iter=10)

  # make output
```

```
  colnames(proj.established2) <- initialPops
  out <- data.frame(prop.good, proj.established2)
  outlist <- list(simList=simList, estab=out, lam.s=sumlam1,
                  lam.s2=sumlam2, lam.more.than.one=more.one,
                  prop.estab2cond=prop.estab2cond)
  return(outlist)
} # end diffInitialCond function
```
